# Pattern of neuropsychological performance among HIV positive patients in Uganda

**DOI:** 10.1186/1471-2377-7-8

**Published:** 2007-04-05

**Authors:** Kevin R Robertson, Noeline Nakasujja, Matthew Wong, Seggane Musisi, Elly Katabira, Thomas D Parsons, Allan Ronald, Ned Sacktor

**Affiliations:** 1 Department of Neurology, University of North Carolina School of Medicine, Chapel Hill, NC, USA; 2 Department of Medicine, Makerere University, Kampala, Uganda; 3 Department of Neurology, University of Virginia, Charlottesville, VA, USA; 4 Center for Creative Technologies, University of Southern California, Marina del Ray, CA, USA; 5 Department of Medicine, University of Manitoba, Winnipeg, MB, Canada; 6 Department of Neurology, Johns Hopkins University School of Medicine, Baltimore, MD, USA

## Abstract

**Background:**

Few studies have examined cognitive functioning of HIV positive patients in sub-Saharan Africa. It cannot be assumed that HIV positive patients in Africa exhibit the same declines as patients in high-resource settings, since there are differences that may influence cognitive functioning including nutrition, history of concomitant disease, and varying HIV strains, among other possibilities. Part of the difficulty of specifying abnormalities in neuropsychological functioning among African HIV positive patients is that there are no readily available African normative databases. The purpose of the current study was to evaluate the pattern of neuropsychological performance in a sample of HIV positive patients in comparison to HIV negative control subjects in Uganda.

**Methods:**

The neuropsychological test scores of 110 HIV positive patients (WHO Stage 2, n = 21; WHO Stage 3, n = 69; WHO Stage 4, n = 20) were contrasted with those of 100 control subjects on measures of attention/concentration, mental flexibility, learning/memory, and motor functioning.

**Results:**

Analysis of covariance (ANCOVA) revealed significant group differences on measures of verbal learning and memory, speed of processing, attention and executive functioning between HIV seropositive and seronegative subjects.

**Conclusion:**

Ugandan patients with HIV demonstrated relative deficits on measures of verbal learning and memory, speed of processing, attention, and executive functioning compared to HIV negative controls. These results from a resource limited region where clades A and D are prevalent are consistent with previous findings in the developed world where clade B predominates.

## Background

Studies done in the developed world in the era prior to highly active antiretroviral therapy (HAART) have shown prevalence rates of dementia ranging from 7.3%–27.3% in patients with advanced infection [1-3]. Despite the high prevalence of HIV in many African countries, very few studies have examined cognitive functioning in these patients. It cannot be assumed that HIV positive patients in Africa exhibit the same declines as patients in high-resource settings, since there are differences that may influence cognitive functioning including nutrition, history of concomitant disease, and varying HIV strains, among other possibilities.

Cognitive impairments are relatively common in HIV infection. The most striking picture is of frank dementia. HIV associated dementia (HAD) afflicts 10–15% of patients with advanced infection in the United States [3], but there is also a high incidence of less severe dysfunction that has been labeled minor cognitive motor dysfunction (MCMD). HIV-associated cognitive impairment is found across all disease stages, with increasing rates of impairment with increasing systemic stage of HIV disease. The areas most often affected include attention/concentration, learning, executive, information processing speed, motor, and psychomotor speed; consistent with damage to subcortical and frontostriatal systems [3-7]. Consequently, HIV+ patients have higher rates of unemployment [8] and difficulty completing instrumental activities of daily living [9].

Access to highly active antiretroviral therapy in Sub-Saharan Africa is increasing. HAART can improve neuropsychological performance in individuals with HIV-associated cognitive impairment in the United States [10,11], but the effect of HAART on individuals with HIV-associated cognitive impairment in Sub-Saharan Africa is largely unknown. As access to AIDS care and highly active antiretroviral therapy (HAART) increases, greater numbers of individuals are receiving care at ambulatory HIV clinics [12]. Studies have shown that HAART can halt the cognitive decline associated with HIV associated dementia, and in some cases may reverse it [10,11,13].

HIV clade B is found in the United States, Europe, and Australia where almost all neuropsychological studies have been completed. In Uganda HIV clades D and A predominate over clade B [14]. Variations in HIV clade could lead to different expression of HIV dementia. Both prevalence and characterization could be altered. A higher or lower prevalence of HIV associated dementia could exist in non B clade endemic areas, due to possible increased neurotropism. The pattern of neurological and neuropsychological deficits could also be altered in non B clade endemic areas. If the underlying neuropathological areas affected are different in non B clade, then the pattern of functional impairments will change.

The purpose of the current study was to evaluate the pattern of neuropsychological performance in a sample of HIV positive patients in comparison to HIV negative control subjects in Uganda.

## Methods

This study was reviewed and approved by the local institutional review board in Uganda prior to study initiation.

Participants: The study took place at two sites in Kampala, Uganda. HIV+ subjects were seen at the Infectious Disease Clinic (IDC) at the Mulago Hospital Complex. This clinic provides care to HIV+ patients at all stages of their illness. It is partially funded by the Academic Alliance Foundation. HIV- subjects were recruited from the AIDS Information Centre (AIC) in Kampala, Uganda. The AIC provides a voluntary counseling and testing centre for individuals who wish to know their HIV infection status. The AIC tests approximately 120 individuals daily of which about 80% are HIV-. Informed consent was obtained from all subjects at both sites.

One hundred ten HIV+ (WHO Stage 2, n = 21; WHO Stage 3, n = 69; WHO Stage 4, n = 20) participants were recruited from the IDC. Eighty-one HIV+ subjects were recruited using a random clustered sample from attendees of the IDC. The remaining subjects were recruited for a pilot study to evaluate the effect of HAART and had CD4 counts < 200. Forty nine of the subjects had a history of antiretroviral treatment with varying regimens. Exclusion criteria were: HIV seronegative, less than 18 years of age, an active or known past CNS opportunistic infection, fever > 37.5°C, a history of a chronic neurological disorder, active psychiatric disorder, alcoholism, physical deficit (e.g., amputation), severe functional impairment (Karnofsky < 50), or severe medical illness that would interfere with the ability to perform the study evaluations.

100 HIV- individuals were recruited from the client base of the AIC. In order to be considered HIV- the individual had to have a negative HIV ELISA test in the last year. HIV- subjects were selected to match the mean age and education of the randomly selected HIV+ sample. Applicable exclusion criteria for the HIV+ subjects were used for the HIV- subjects.

The 110 HIV positive participants had mean age in years of 36.72 (SD = 8.71) ; education in years of 9.11 (SD = 5.02), and most were female (69.57%). The 100 HIV negative control participants had mean age in years of 27.48 (SD = 9.14), education in years of 12.08 (SD = 4.05) and 40 percent female.

### WHO Disease Staging System

Given the fact that our research was conducted in the African Region, we utilized the World Health Organization's (WHO) clinical staging and case definition of HIV in resource-constrained settings. The WHO disease staging system for HIV Infection was first produced in 1990 and updated in September 2005 (World Health Organization, 2005). WHO clinical stages are categorized as Stage 1 through Stage 4, and reflect progression from primary HIV infection to advanced HIV/AIDS. Each stage is defined by specific clinical conditions or symptoms: In Stage 1, HIV disease is asymptomatic and not categorized as AIDS. In Stage 2: minor mucocutaneous manifestations and recurrent upper respiratory tract infections are included. In Stage 3, unexplained chronic diarrhea (longer than a month), and severe bacterial infections and pulmonary tuberculosis are included. In Stage 4, diseases used as indicators of AIDS (brain toxoplasmosis, candidiasis of the esophagus, trachea, bronchi or lungs, and Kaposi's sarcoma) are included.

### Instruments

Data collection included five sections: (1) A sociodemographic survey, (2) medical history and functional status survey, (3) subjective neurological symptoms questionnaire, (4) neurological assessment, and a (5) neuropsychological test battery.

The neuropsychological instruments administered to both HIV+ and HIV- patients were chosen for their cultural independence as well as their sensitivity for detecting HIV dementia in HIV+ patients [15]. The tests include assessments in the domains of functioning of Gross motor (Timed Gait), Fine motor (Grooved pegboard), Executive Functioning (Color Trails 2), Speed of Processing (Color Trails 1, Symbol Digit modalities), Verbal Learning/Memory (WHO-UCLA Auditory Verbal Learning Test), and Attention/Working Memory (Digit Span, Forward and Backward).

The WHO-UCLA Auditory Verbal Learning Test is similar to the Rey Auditory Verbal Learning Test [16,17]. These tests both measure verbal learning and memory through a scored immediate and delayed recall of a 15 word list. While the structure of each test is the same, the words used in the WHO-UCLA test have been chosen to be universally recognized objects independent of culture and language [18]. Color Trails 1 and 2 test an individual's speed of processing and are designed as cross-cultural variants of the Trail Making tests [16,18-20].

All the tests had their instructions and content translated into Luganda. Tests were administered in either Luganda or English, depending on a formal assessment of the main language of the subject. Test administrators were fluent in both languages. Most of the subjects (81% HIV- and 77% HIV+) were tested in Luganda. An ANCOVA found no difference for main language, and no significant differences for main language within HIV groups on the neuropsychological tests when years of education was adjusted for. Scores for the individual tests were summed then averaged into a Total z-score. Note that, we multiplied the Timed Gait, Grooved pegboard, Color Trails 1 & 2, and Symbol Digit modalities scores by (-1) so that all positive z-scores scores indicate improvement and negative scores denote a decline in performance.

### Determination of Dementia

Each HIV+ individual had their demographics, past medical history, neurological symptoms, functional status, neuropsychological test scores and neurological exam assessed by the primary examiners, an HIV neurologist and an HIV neuropsychologist in a consensus conference. Using this information a Memorial Sloan-Kettering (MSK) AIDS dementia stage score of 0 (MSK normal or no cognitive impairment), 0.5 (MSK subclinical), and greater than or equal to 1 (MSK dementia) was assigned to each HIV+ individual [21]. In order to be given a diagnosis of HIV AIDS dementia stage (MSK score = 1 or greater), a patient had to be impaired in two separate neuropsychological domains, as well as have significant functional impairment.

### Data Analytic Plan

HIV+ subjects were stratified based on MSK AIDS Dementia stage (0, 0.5 and greater than 1.0). To explore differences on cognitive domains between groups analysis of variance was utilized. To examine the relationship between continuous variables of interest Spearman correlations were calculated. Statistical analysis was done in SAS 9.1.3 (SAS Institute Inc., Cary, NC). Analysis of Covariance was utilized where appropriate.

## Results

Using ANCOVA, there was a significant difference in neuropsychological total z-scores between the HIV seronegatives and seropositives (F (2, 209) = 32.36, p < .0001) after adjusting for differences in education. Similarly, as shown in Table 1, ANCOVA adjusting for education found significant differences between HIV-and HIV+ subjects for AVLT total, AVLT Delayed Recall, Symbol Digit, Color Trails 1, Color Trails 2, Digit Span Forward and Digit Span Backward. Modified Timed Gait, Grooved Pegboard using the dominant and nondominant hands did not reach significance between HIV- and HIV+ subjects after adjusting for education.

There were significant differences found in neuropsychological tests between MSK AIDS Dementia Stage (see Table 2). As expected, as AIDS Dementia Stage increased, poorer outcomes on the neuropsychological tests were found for the total z score (p < .0001), AVLT Total (p < .0001), Delayed Recall (p < .0001), Symbol Digit (p < .0001), Grooved Pegboard Dominant (p < .001), Nondominant (p < .005), Timed Gait (p < .0005), Color Trails 1 and 2 (p < .0001, both), and Digit Span backward (p < .001) and forward (p < .06). On most neuropsychological tests, post hoc analysis found that MSK normal group was equivalent to MSK subclinical group, and MSK dementia group had poorer functioning than both groups. On Symbol Digit and Color Trails 2, the MSK normal group was significantly better than the MSK subclinical group which was significantly better than those with MSK dementia, providing the most separation between groups. On Timed Gait, MSK normal was better than the MSK Subclinical group, which was equivalent to the MSK mild dementia group.

## Discussion

HIV dementia in clade B occurs in up to 27% of patients with advanced HIV prior to effective treatment and has been associated with CNS white matter disease, often affecting speed of processing and motor functioning. [1-3]. We have previously reported a cross sectional HIV dementia rate of 31% in this same group from Uganda presumably with subtype A and D [22].

Here, we assessed performance between HIV seronegative controls and HIV seropositives in Uganda where HIV clades D and A predominate over clade B [14]. Variations in HIV clade could lead to differences in prevalence rates, and/or differences in the functional deficit pattern of HIV Dementia. Increased neurotropism relative to clade B would lead to a higher prevalence of AIDS Dementia in non B clade endemic areas, while decreased neurotropism would result in lower prevalence rates. Similarly, differences in the underlying neuropathological areas affected across clades would result in changes in the pattern of neurological and neuropsychological functional impairments. For example, a greater preference for frontal areas in one clade, could result in larger deficits in executive functioning for those affected. Our primary objective was to characterize the pattern of neuropsychological functioning in this Ugandan cohort.

In Uganda, a setting of predominantly HIV clade D and A in sub-Saharan Africa, our study found significant group differences between the HIV seronegatives and HIV seropositives on measures of verbal learning and memory, speed of processing, attention and executive functioning. As HIV disease stage progressed, correlations with poorer verbal learning and memory, speed of processing, attention/working memory, and executive function were found. Characterization of neuropsychological performance across MSK AIDS Dementia Stage found that all tests were poorer in those with MSK AIDS Dementia compared to those without. Executive function, Speed of processing, and Gross Motor functioning were poorer in those with MSK Subclinical Dementia than those HIV+ individuals with no evidence of dementia.

HIV+ Ugandans demonstrated a relative decline, in measures of verbal learning and memory, speed of processing, attention, and executive functioning compared to HIV negative controls. This is a similar pattern to what has been found in the US, Europe and Australian settings, and would suggest that the pattern of neuropsychological loss will be the same with clade A and D HIV infection [5,15]. In turn, this would suggest that HIV clade A and D have the same underlying pattern of neuropathology. There was no formal assessment of clade in this study. However, another study found faster systemic progression in clade D over clade A in Uganda. [23] Given that neuropsychological deficits are often correlated to immunological deficits and systemic disease progression, one would expect higher deficits in those with clade D compared to clade A over time.

We found no significant difference in the fine and gross motor tests between the HIV positive and HIV negative groups in this setting. However, in this study we found that both fine and gross motor performance become more impaired in those with AIDS Dementia, and may be useful in separating those with Subclinical Dementia from normals. In a separate study using the International HIV Dementia Scale, (IHDS), a screening test for HIV dementia which evaluates motor and psychomotor speed performance, we have found that motor functioning remains important in determining AIDS Dementia Stage in these Ugandan patients [24]. Another study on cognitive functioning in a non-clade B HIV sample has suggested that other HIV clades are neuropathogenic. Cognitive functioning in individuals infected with clade C in India found neuropsychological deficits in HIV+ subjects compared to HIV- subjects [25].

AIDS related neurological disease has a very high cost in terms of loss of functional ability. Patients have higher rates of unemployment, decreased ability to perform their activities of daily living including difficulty completing such tasks as meal preparation and housework [8,26]. These data suggest that Ugandan patients demonstrate a relative decline on measures of verbal learning and memory, speed of processing, attention, and executive functioning compared to HIV negative controls. These deficits are likely to have an impact on a patient's ability to maintain employment outside of the home, or to complete activities to maintain the household. When this happens, many others are affected as well. Dependent children or parents will also experience the losses associated with these deficits: less income, food and caretaking ability. Initiation of antiretroviral therapy in these individuals may reverse some of these neuropsychological deficits, and allow continued employment and household maintenance.

There are noteworthy limitations that should be considered when interpreting the current study's findings. While there is a need for neurocognitive assessments in resource limited settings, our battery was relatively brief, which may limit its sensitivity to HIV-related neurocognitive impairment [27]. Further, we suspect that speaking Luganda is a surrogate marker for multiple factors, such as lower socioeconomic status, decreased access to healthcare and decreased nutritional status. These factors were not measured in the current study, but inclusion of these factors in future work may determine any association with an increased risk of HIV dementia. Control subjects were determined by requiring the negative control subjects to have a negative HIV ELISA in the last year. There is a chance that some control subjects could have become HIV positive within that time frame.

Further studies to characterize HIV associated dementia and neuropsychological functioning in resource limited settings are necessary. The response of neurological disease to treatment will also be important to characterize. These studies will be particularly difficult to engage in, but can be aided by committed researchers on the ground that are competent and bring the necessary local experience to successfully complete them.

## Conclusion

The current study found neuropsychological deficits in a region where clades A and D are prevalent, consistent with previous findings in the developed world where clade B predominates. Ugandan patients with HIV demonstrated deficits on measures of verbal learning and memory, speed of processing, attention, and executive functioning compared to HIV negative controls. Fine and gross motor performance became more impaired in those with AIDS Dementia. The pattern of neuropsychological deficits found in this study is similar to what has been found in the US, Europe, and Australia, suggesting that HIV clades A and D have the same underlying neuropathology as clade B. These neuropsychological deficits are likely to influence the patient's activities of daily living and ability to maintain employment, although initiation of antiretroviral therapy has been shown to improve these deficits in some.

## Competing interests

The author(s) declare that they have no competing interests.

## Authors' contributions

KR supervised the neuropsychological aspects of the study, carried out the statistical analyses, participated in conception of the study design and drafted the manuscript. NN participated in conception of the study design, supervised and carried out the neurological and neuropsychological exams, and revised the manuscript. MW participated in conception of the study design, supervised and carried out the neurological and neuropsychological exams, SM participated in study design, supervised and carried out the neurological and neuropsychological exams. EK provided supervision of the overall study, including neurological and neuropsychological exams. TP participated in the statistical analyses and revisions of the manuscript. AR participated in the study design, overall supervision and funding of the study. NS supervised the neurological and neuropsychological aspects of the study, participated in conception of the study design and funding and revisions the manuscript. All authors read and approved the final manuscript.

## Sponsorship

Supported by Bill and Melinda Gates Foundation and the Academic Alliance Foundation (which has received support from Pfizer Pharmaceuticals). MH62690, AI25868, AI50410, RR00046. Further support for this work: RO1NSMH34243, RO1MH71150.

The funding bodies above had no role in study design; in the collection, analysis, and interpretation of data; in the writing of the manuscript; or in the decision to submit the manuscript for publication.

## Pre-publication history

The pre-publication history for this paper can be accessed here:



## Figures and Tables

**Table 1:** Descriptives of Neuropsychological Functioning of HIV seronegatives and HIV seropositives.

	**HIV seropositives**			**HIV seronegatives**				
	**Valid N**	**Mean**	**SD**	**Valid N**	**Mean**	**SD**	**F**	**p**
AVLT Recall	109	7.25	2.48	100	9.2	2.27	32.63	< .0001
AVLT Total	110	37.25	8.66	100	43.57	7.41	28.81	< .0001
Symbol Digit	105	24.13	11.41	100	31.12	11.27	18.96	< .0001
Pegboard Dom	108	91.58	26.9	100	86.48	21.33	1.19	ns
Pegboard Non	108	102.78	37.52	100	102.71	25.23	0.17	ns
Gait	108	7.21	1.65	98	6.95	0.82	1.2	ns
Trails 1	107	92.67	49.71	99	74.38	22.21	9.65	< .0005
Trails 2	106	166.97	57.67	99	124.51	37.01	33	< .0001
Digit forward	110	4.91	0.89	100	5.28	0.87	7.36	< .005
Digit back	110	2.99	1.1	100	3.47	0.9	8.48	< .001

AVLT = WHO-UCLA Auditory Verbal Learning Test; Dom = Dominant hand; Non = nondominant hand; Gait = Modified Timed Gait; Trails = Color Trails; Digit forward = WAISR Digit Span Forward; Digit Back = WAISR Digit Span Backward.

**Table 2:** Neurocognitive performance by ADC Stage

	**0 (Normal)**		**0.5 (subclinical)**		**1 (mild)**				
	**Mean**	**SD**	**Mean**	**SD**	**Mean**	**SD**	**F**	**p**	**Group difference**
Total z	0.30	0.32	-0.29	0.48	-1.26	.78	44.16	0.0001	0 > .5 > 1
AVLT Recall	8.84	1.71	8.14	2.23	5.5	2.06	16.99	0.0001	0 = .5 < 1
AVLT Total	43	5.52	40.7	7.7	33.8	6.18	11.77	0.0001	0 = .5 < 1
Symbol Digit	36.63	7.85	25.77	10.78	17.33	9.81	20.36	0.0001	0 > .5 > 1
Pegboard Dom	78.47	8.44	86.73	21.62	106.2	35.69	7.55	0.001	0 = .5 > 1
Pegboard Non	86.53	9.43	93.97	18.68	122.68	64.06	6.08	0.005	0 = .5 > 1
Gait	6.6	0.71	6.82	0.71	7.64	1.22	8.85	0.0005	0 < .5 = 1
Trails 1	60.47	20.87	81.43	39.08	117.56	52.68	11.35	0.0001	0 = .5 < 1
Trails 2	109.11	23.27	159.77	46.64	216.08	55.56	30.23	0.0001	0 < .5 < 1
Digit forward	5.26	0.99	5.03	0.83	4.64	0.81	2.99	0.06	0 = .5,.5 = 1, 0 < 1
Digit back	3.79	0.85	3.08	1.06	2.64	0.86	7.79	0.001	0 < .5 = 1

ADC Stage is the Memorial Sloan Kettering AIDS Dementia Staging

## References

[B1] Janssen RS, Nwanyanwu OC, Selik RM, Stehr-Green JK (1992). Epidemiology of human immunodeficiency virus encephalopathy in the United States. Neurology.

[B2] Portegies P, Enting RH, de Gans J, Algra PR, Derix MM, Lange JM (1993). Presentation and course of AIDS dementia complex: 10 years of follow-up in Amsterdam, The Netherlands. AIDS.

[B3] Sacktor N (2002). The epidemiology of human immunodeficiency virus-associated neurological disease in the era of highly active antiretroviral therapy. J Neurovirol.

[B4] Saykin AJ, Janssen RS, Sprehn GC, Kaplan JE, Spira TJ, O'Connor B (1991). Longitudinal evaluation of neuropsychological function in homosexual men with HIV infection: 18-month follow-up. J Neuropsychiatry Clin Neurosci.

[B5] Heaton RK, Grant I, Butters N, White DA, Kirson D, Atkinson JH, McCutchan JA, Taylor MJ, Kelly MD, Ellis RJ (1995). The HNRC 500 – neuropsychology of HIV infection at different disease stages. HIV Neurobehavioral Research Center. J Int Neuropsychol Soc.

[B6] McArthur JC, Haughey N, Gartner S, Conant K, Pardo C, Nath A, Sacktor N (2003). Human immunodeficiency virus-associated dementia: an evolving disease. J Neurovirol.

[B7] Tozzi V, Balestra P, Lorenzini P, Bellagamba R, Galgani S, Corpolongo A, Vlassi C, Larussa D, Zaccarelli M, Noto P, Visco-Comandini U, Giulianelli M, Ippolito G, Antinori A, Narciso P (2005). Prevalence and risk factors for human immunodeficiency virus-associated neurocognitive impairment, 1996 to 2002: results from an urban observational cohort. J Neurovirol.

[B8] Albert SM, Marder K, Dooneief G, Bell K, Sano M, Todak G, Stern Y (1995). Neuropsychologic impairment in early HIV infection. A risk factor for work disability. Archives of Neurology.

[B9] Benedict RH, Mezhir JJ, Walsh K, Hewitt RG (2000). Impact of human immunodeficiency virus type-1-associated cognitive dysfunction on activities of daily living and quality of life. Arch Clin Neuropsychol.

[B10] Sacktor NC, Lyles RH, Skolasky RL, Anderson DE, McArthur JC, McFarlane G (1999). Combination antiretroviral therapy improves psychomotor speed performance in HIV-seropositive homosexual men. Multicenter AIDS Cohort Study (MACS). Neurology.

[B11] Sacktor NC, Skolasky RL, Lyles RH, Esposito D, Selnes OA, McArthur JC (2000). Improvement in HIV-associated motor slowing after antiretroviral therapy including protease inhibitors. Journal of Neurovirology.

[B12] World Health Organization (2004). '3 by 5' Progress Report.

[B13] Robertson KR, Robertson WT, Ford S, Watson D, Fiscus S, Harp AG, Hall CD (2004). Highly active antiretroviral therapy improves neurocognitive functioning. J Acquir Immune Defic Syndr.

[B14] Kaleebu P, Whitworth J, Hamilton L, Rutebemberwa A, Lyagoba F, Morgan D, Duffield M, Biryahwaho B, Magambo B, Oram J (2000). Molecular epidemiology of HIV type 1 in a rural community in southwest Uganda. AIDS Res Hum Retroviruses.

[B15] Miller EN, Selnes OA, McArthur JC, Satz P, Becker JT, Cohen BA, Sheridan K, Machado AM, Van Gorp WG, Visscher B (1990). Neuropsychological performance in HIV-1-infected homosexual men: The Multicenter AIDS Cohort Study (MACS). Neurology.

[B16] Maj M, D'Elia L, Satz P, Janssen R, Zaudig M, Uchiyama C, Starace F, Galderisi S, Chervinsky A (1993). Evaluation of two new neuropsychological tests designed to minimize cultural bias in the assessment of HIV-1 seropositive persons: a WHO study. Arch Clin Neuropsychol.

[B17] Rey A (1941). L'examen psychological dans les cas d'encephalopathie traumatique. Archives of Psychology.

[B18] Maj M, Satz P, Janssen R, Zaudig M, Starace F, D'Elia L, Sughondhabirom B, Mussa M, Naber D, Ndetei D (1994). WHO Neuropsychiatric AIDS study, cross-sectional phase II. Neuropsychological and neurological findings. Arch Gen Psychiatry.

[B19] Dugbartey AT, Townes BD, Mahurin RK (2000). Equivalence of the Color Trails Test and Trail Making Test in nonnative English-speakers. Archives of Clinical Neuropsychology.

[B20] Reitan R (1958). Validity of the Trail Making test as an indicator of organic brain damage. Perceptual and Motor Skills.

[B21] Price RW, Brew BJ (1988). The AIDS dementia complex. Journal of Infectious Diseases.

[B22] Wong MH, Robertson K, Nakasujja N, Skolasky R, Musisi S, Katabira E, McArthur JC, Ronald A, Sacktor N (2007). Frequency of and risk factors for HIV dementia in an HIV clinic in sub-Saharan Africa. Neurology.

[B23] Laeyendecker O, Li X, Arroyo M, McCutchan F, Gray R, Wawer M, Serwadda D, Nalugoda F, Kigozi G, Quinn T (2006). The effect of HIV subtype on rapid disease progression in Rakai, Uganda. 13th Conference on Retroviruses and Opportunistic Infections.

[B24] Sacktor NC, Wong M, Nakasujja N, Skolasky RL, Selnes OA, Musisi S, Robertson K, McArthur JC, Ronald A, Katabira E (2005). The International HIV Dementia Scale: a new rapid screening test for HIV dementia. Aids.

[B25] Yepthomi T, Paul R, Vallabhaneni S, Kumarasamy N, Tate DF, Solomon S, Flanigan T (2006). Neurocognitive consequences of HIV in southern India: a preliminary study of clade C virus. J Int Neuropsychol Soc.

[B26] Mindt MR, Cherner M, Marcotte TD, Moore DJ, Bentley H, Esquivel MM, Lopez Y, Grant I, Heaton RK (2003). The functional impact of HIV-associated neuropsychological impairment in Spanish-speaking adults: a pilot study. J Clin Exp Neuropsychol.

[B27] Smith CA, van Gorp WG, Ryan ER, Ferrando SJ, Rabkin J (2003). Screening subtle HIV-related cognitive dysfunction: the clinical utility of the HIV dementia scale. J Acquir Immune Defic Syndr.

